# Revolutionizing Dental Care: A Comprehensive Review of Artificial Intelligence Applications Among Various Dental Specialties

**DOI:** 10.7759/cureus.47033

**Published:** 2023-10-14

**Authors:** Najd Alzaid, Omar Ghulam, Modhi Albani, Rafa Alharbi, Mayan Othman, Hasan Taher, Saleem Albaradie, Suhael Ahmed

**Affiliations:** 1 Dentistry, University of Hail College of Dentistry, Hail, SAU; 2 General Dentistry, Prince Mohammed bin Abdulaziz Hospital, Madinah, SAU; 3 Dentistry, Taibah University College of Dentistry, Madinah, SAU; 4 Endodontics, Prince Mohammed bin Abdulaziz Hospital, Madinah, SAU; 5 Maxillofacial Surgery and Diagnostic Sciences, College of Medicine and Dentistry, Riyadh Elm University, Riyadh, SAU

**Keywords:** artificial intelligence in dentistry, endodontic infections, dentistry related articles, digital dentistry, ai

## Abstract

Since the beginning of recorded history, the human brain has been one of the most intriguing structures for scientists and engineers. Over the centuries, newer technologies have been developed based on principles that seek to mimic their functioning, but the creation of a machine that can think and behave like a human remains an unattainable fantasy. This idea is now known as "artificial intelligence". Dentistry has begun to experience the effects of artificial intelligence (AI). These include image enhancement for radiology, which improves the visibility of dental structures and facilitates disease diagnosis. AI has also been utilized for the identification of periapical lesions and root anatomy in endodontics, as well as for the diagnosis of periodontitis. This review is intended to provide a comprehensive overview of the use of AI in modern dentistry's numerous specialties. The relevant publications published between March 1987 and July 2023 were identified through an exhaustive search. Studies published in English were selected and included data regarding AI applications among various dental specialties. Dental practice involves more than just disease diagnosis, including correlation with clinical findings and administering treatment to patients. AI cannot replace dentists. However, a comprehensive understanding of AI concepts and techniques will be advantageous in the future. AI models for dental applications are currently being developed.

## Introduction and background

History

Over the years, numerous medical imaging techniques, such as X-ray, mammography, computed tomography (CT), magnetic resonance imaging (MRI), and ultrasound, have significantly contributed to the effective diagnosis and treatment of numerous diseases [1]. However, as burdens and complexities increase, human experts may experience fatigue, which could compromise the precision of their results [2]. Over a period of time, newer technologies have been developed, but the creation of a machine that can think and function like a human remains an unattainable fantasy, which is now referred to as "artificial intelligence" (AI) [3]. AI is a discipline of computer science and engineering that concentrates on the computational comprehension of what is commonly referred to as intelligent behavior and the development of intelligent artifacts. The human brain is a unique structure made up of networks of interconnected neurons that transmit signals throughout the entire body [4]. AI systems range from expert systems to those that use intricate computational models to predict new information by learning from data. The latter category contains machine learning (ML) systems that employ a wide variety of tools, techniques, and algorithms [5]. ML is a type of AI algorithm and model that is "trained" to recognize statistical patterns in a given data set (known as the training data) in order to recognize similar patterns in new data (known as the test data) [6]. These models have demonstrated high accuracy and sensitivity. AI has begun to have varied effects on the discipline of dentistry [7]. These include image enhancement for radiology, which improves the visibility of dental structures and facilitates the diagnosis of cysts and tumors [8-11]. AI has also been utilized for the determination of periapical lesions and root anatomy for endodontics [12-16], as well as for the diagnosis of periodontitis [14-19]. AI has also been used to automate the identification of cephalometric landmarks in orthodontics. Several investigations have supported these developments. As technology continues to advance, the field of surgery, from ophthalmology [10], to spinal surgery [11], to knee arthroplasty [12], has extensively explored the use of AI. AI in dentistry is a "work in rapid progress".

Alan Turing, a British mathematician, in 1936, demonstrated the feasibility of a universal calculator, also known as the "Turing machine", which was capable of solving any problem that could be represented and solved using an algorithm [13]. The first AI program, "The Logic Theorist", which Newell and Simon produced in 1955, signaled the start of the modern AI era. McCarthy subsequently coined the term "artificial intelligence" in 1956 [14].

Currently, ML is applied to the analysis of dental radiographs and images [15]. AI is currently used to analyze X-rays and cone beam computed tomography (CBCT) imaging, detect periodontal diseases, predict treatment outcomes, and diagnose oral malignancies [16].

The aim of the study

The purpose of this review was to provide a comprehensive overview of the use of AI in various specialties in modern dentistry.

Materials and methods

Pubmed, Google Scholar, Scopus, Web of Science, Embase, Cochrane, and the Saudi Digital Library were used to conduct a comprehensive search for relevant articles published between March 1987 and April 2023. By manually examining the articles and their references, a search was conducted. Studies were chosen if they were written in English and had information about dental caries, tooth decay, cavities, diagnosis, detection, prediction, restorative, orthodontics, oral cancer, oral disease, periodontal disease, and endodontics, as well as their relationship to AI, ML, deep learning (DL), automated systems, convolutional neural networks (CNNs), artificial neural networks (ANNs), and deep convolutional neural networks (DCNNs).

## Review

Terms in AI

**Figure 1:**
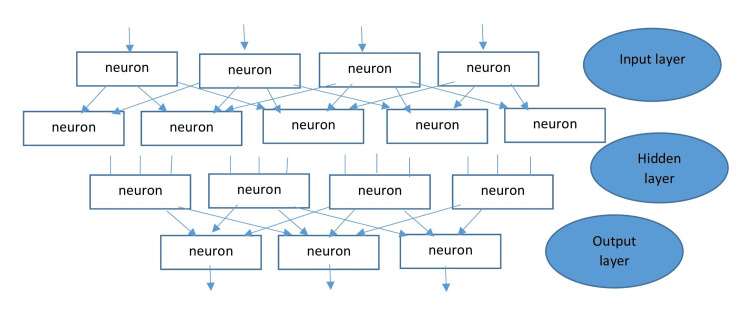
A feedforward multilayer neural network

The subsets of AI include ML, neural networks, and DL. Without human intervention, ML algorithms can solve prediction problems through data-driven learning. Neural networks are created to imitate the non-linear mathematical model of the human brain by employing artificial neurons that simulate human cognitive abilities such as problem-solving, decision-making, and learning. A rudimentary neural network consists of an input layer where data is input, a concealed layer where data is processed, and an output layer where the system makes decisions. Neural networks can map any input to any output, given a set of mathematical models and sufficient data to represent the intrinsic statistical figures of the input data. Figure 1 from Swietlik D et al. depicts the topology of a straightforward ANN [17].

To discover the relationship between the features and the ground truth, the AI algorithm may employ techniques such as random forests, support vector machines, or ANNs [20]. ANNs mimic the biological neurons in the human brain, which have multiple adaptive connections during learning. They consist of neurons (nodes) that use ML algorithms to recognize patterns and connections in a dataset, analogous to the data processing function of the brain [21].

In healthcare, there are two categories of AI: virtual and physical. Virtual AI employs mathematical algorithms for diagnosis, prognosis, imaging, appointment scheduling, drug dosage calculations, drug interaction assessments, and electronic health records [19]. Physical AI encompasses the use of robots in surgical assistance [22], telepresence [23], rehabilitation, and care for the elderly [24].

Multilayer perception (MLP) neural networks are more complex ANNs with additional hidden layers. The commonly used types of neural networks include ANNs, CNNs, and recurrent neural networks. DL is a subset of neural networks where the computer can learn to process data on its own. DL neural networks have a large number of neurons in the hidden layer, ranging from a few thousand to several million. These concepts have been extensively studied and researched and have been reported in several scientific articles [18-22].

ML is a subfield of computer science that involves the construction of algorithms using data as a guide. DL is a specialized form of learning based on neural network algorithms.

DL refers to a specific type of learning that uses neural network-based algorithms.

ANNs are artificial neural networks. It is a system of interconnected computer processors that can learn from previous instances, analyze nonlinear data, deal with uncertain information, and make generalizations, making it a highly desirable analytical method in the medical field.

CDSS is a clinical decision support system. This refers to a system that combines an extensive database of medical knowledge with algorithms based on evidence-based medical practices. It employs medical logic modules to draw conclusions and generate output. The system also features a user-friendly interface with voice-activated controls, which are intended to help healthcare professionals work more effectively, save time, and provide cost-effective dental care.

The term enhanced reality refers to a technology that combines the user's real-world view with a computer-generated image to create a composite view.

Simulated reality - a virtual reality - is a computer-simulated 3D image or environment that can be interacted with using specialized electronic equipment in a manner that appears real and physical.

E-learning is an important aspect of dentistry that emphasizes skill development and patient care improvement. In the past, pre-clinical operative training for dental students involved a combination of theoretical teaching and practical learning. However, augmented and virtual reality technologies have been incorporated into dental education to create intelligent tutoring and training systems. These technologies provide access to clinical and surgical procedures and allow for the simulation of practical procedures in three dimensions. By practicing in a simulated environment, students are able to repeat their practice sessions until they are proficient in the subject, thereby reducing the risk of iatrogenic error when handling actual clinical cases.

Developments and applications in various dental specialties

AI in Prosthodontics

AI can assist dentists in designing aesthetically appealing and functional prostheses by integrating with design software and taking into account factors such as facial dimensions, anthropological calculations, ethnicity, and patient preferences. AI is also instrumental in detecting bone types and cortical thickness for precise positioning of implants using surgical guides [25]. Dr. Werner Mormann and Marco Brandestini introduced the first CAD/CAM system to a dental clinic at the University of Zurich in the 1980s, marking the beginning of AI integration into dentistry. This system has revolutionized dental practice by allowing dentists to generate multiple ceramic restorations in clinics and laboratories. By scanning a patient's remaining teeth, CAD/CAM technology enables the creation of 3D models of dental crowns that are customized to the patient's preferences. The models can then be printed and cast with ease, expediting the manual process of producing prostheses [26]. CAD/CAM technology replaces the time-consuming and labor-intensive traditional casting process and reduces the likelihood of human error [27]. Virtual reality simulation (VRS) technology can be used to simulate post-treatment facial profiles, enabling dentists to efficiently design aesthetics and serving as a motivational tool for patients [28].

AI in Restorative Dentistry

Traditionally, dental caries diagnosis involves visual inspection and radiographic assessment. However, the dependability and precision of this method can differ greatly, especially depending on the dentist's clinical experience. Sensitivity can range between 19% and 92% for occlusal caries and between 39% and 94% for proximal caries [29]. Radiographic parameters like shadow, contrast, and luminance may have an impact on the diagnosis. Extensive research has been conducted on AI models for predicting, detecting, and diagnosing dental caries. These models have exhibited outstanding performance and can be used in clinical practice to identify patients at increased risk for dental caries and improve diagnosis, treatment quality, and patient outcomes [30]. DCNN can be used to make a dental caries detector that can learn and identify the location and shape of dental caries lesions. This makes it a reliable and useful diagnostic tool [31,32]. A study by Devito et al. evaluated an AI model for diagnosing proximal caries from bitewing radiographs and discovered that the AI software had greater diagnostic accuracy than the most experienced human examiner, as confirmed by histologic analysis of extracted human teeth [33]. The reviewed studies demonstrated that AI models have an accuracy range of 76% to 88.3% for diagnosing caries, a sensitivity range of 73% to 90%, and a specificity range of 61.5% to 93%. However, only one study [34], using near-infrared transillumination imaging, made a CNN-based AI model for diagnosing dental caries. It had an accuracy of 72% and better results for diagnosing dentin caries than enamel caries. To measure the dimensions of cavitated tooth surfaces, only one study utilized a fiber optic displacement sensor [FODS] [34]. Yamaguchi et al. developed an AI CNN model to predict composite resin crown debonding. In their study, they collected information on 24 composite resin crowns and their virtual dental preparation die files, of which half had debonding issues. They used 640 2D images of 3D virtual dental preparation dies to establish a correlation with debonding data. Using only tooth preparation morphology as a variable, the AI model predicted debonding failures with a 98.5% accuracy rate. Other factors that may have influenced debonding and restoration failure, such as the clinical condition of the tooth, cement selection, and cementation protocol, were not considered. Aliaga and coworkers developed a case-based learning model to determine the optimal restorative material (composite resin or amalgam) for direct dental restorations [35]. Data from 2023 patients, including information on the tooth receiving the restoration and patient characteristics, was collected. The study demonstrated that the model accurately predicted the most appropriate restoration type for each patient by estimating the restoration's longevity. The use of neural networks in conservative dentistry has developed rapidly, but its application is still limited [36]. Algorithms have been created to identify anatomical and pathological structures, which can be difficult due to image noise and low contrast. Geetha et al. used an ANN to detect caries in 105 radiograph images in their study. To distinguish between caries and healthy teeth, the researchers used 16 feature vectors extracted from the segmented image as input nodes and two output nodes. The detection of caries was 97.1% accurate, with a 2.8% false-positive rate. This study demonstrates that neural networks may detect tooth degeneration more accurately than conventional dental examinations [37]. AI has numerous applications, including the identification and categorization of dental restorations. Abdalla-Aslan et al. conducted a study in 2020 in which they used algorithms to find 93.6% of dental restorations on 83 panoramic images and put them into 11 categories based on their shape and gray value distribution [38]. Another study found that an ANN can predict the optimal excavation method for an individual patient with an accuracy of 99.03%, which was microbiologically verified. Using the Logic on Caries Detector, AI can also assist with the detection and classification of proximal caries. Moreover, AI can analyze the lifecycle of various restorative materials and assist in selecting the most suitable material for specific instances [39].

AI in Periodontal Disease

Periodontitis is an extremely prevalent disease worldwide. It is characterized by inflammation, which is a result of both pathogenic factors and the host's immune response and ultimately leads to bone and periodontal attachment loss, which can result in tooth loss [40]. This condition is well documented, but its interaction with various physiological systems is complex, resulting in detrimental effects on life quality and overall health [41]. In addition, studies have demonstrated a bidirectional relationship between periodontitis and systemic conditions such as chronic inflammatory diseases such as diabetes [42,43] and atherosclerosis [44]. Recognizing and diagnosing periodontitis is a challenging task for clinicians [45]. Current best practices predominantly involve the use of a graduated instrument to measure soft tissues [46] and radiographic imaging to evaluate hard tissues [47]. Inconsistent penetrating pressure and changes in radiographic angle make these methods less reliable, which makes it hard for operators to agree with each other [48]. On the other hand, AI has demonstrated promise for improving the detection of periodontitis, including its various types and its relationship to systemic diseases. Lee et al. [49] conducted a study using a computer-aided diagnosis (CAD) system with a DCNN algorithm to diagnose and predict teeth with periodontal health problems. The CNN algorithm demonstrated a range of 76.7% to 81.0% accuracy in diagnosing periodontally compromised teeth (PCTs) and 73.4% to 82.2% accuracy in predicting the need for tooth extraction. Notably, there were variations in accuracy between tooth types, with premolars being diagnosed as PCTs more accurately than molars (82.8% versus 73%). Premolars typically have a single root, whereas molars typically have two or three roots, creating a more complex anatomical structure for the CNN algorithm to understand and explain this discrepancy. Yauney et al. [48] employed an AI system based on a CNN to establish connections between poor periodontal health and systemic health outcomes. Their results suggested that AI could facilitate automated disease diagnosis and serve as a valuable screening instrument for other diseases. In a different study, Papantanopoulos et al. [49] looked at immunologic parameters like leukocytes, interleukins, and IgG antibody titers to tell the difference between aggressive and chronic periodontitis in patients using an ANN. In correctly categorizing patients, the accuracy of the ANN ranged between 90% and 98%. Notably, the ANN that included inputs such as monocyte, eosinophil, and neutrophil counts, as well as the CD4+/CD8+ T-cell ratio, provided the most accurate overall prediction. The study found that ANNs could accurately tell the difference between aggressive and chronic periodontitis using relatively simple and easy-to-get parameters, such as peripheral blood leukocyte counts. Wang et al. devised a system based on the architecture of a digital CNN with 16 convolution layers and two entirely connected layers. This system was designed particularly to detect periodontitis in the premolars and molars [50]. Krois et al. utilized CNNs to detect periodontal bone loss by analyzing panoramic dental radiographs [51] using CNNs. DL analysis of radiographs has shown that it has the potential to make it easier to diagnose and plan treatment for periodontal diseases by making it possible to find early changes in the gums [52,53]. This early intervention is especially beneficial in the field of implantology. In addition to enhancing our comprehension of periodontitis, this technology facilitates the integration of conventional indicators, immunologic factors, and microbiological parameters into the diagnosis of periodontal conditions [54].

AI in Endodontics

AI has mostly been used in endodontics in a virtual way, especially to find periapical lesions, crown and root fractures, working length, and morphology [55].

Work Duration Determinations

It is crucial for the efficacy of endodontic treatment to determine the exact working length. Radiographic methods, the paper point method, digital tactile sensation, and patient response to a file are employed for this purpose. The cement-dentinal junction (CDJ) is the optimal location for cleaning and filling root canals, as it indicates the commencement of the periodontal ligament and the end of the pulp, according to a study [56]. Nevertheless, the CDJ cannot be identified radiographically [57]. Although there is limited evidence to support the use of digital and patient response techniques for determining the working length in endodontic treatment, radiographic methods are widely used [58]. Electronic apex locators are useful for locating the apical foramen during root canal procedures [59], whereas CBCT is a new adjunct used for this purpose [60]. Digital radiography has benefits such as reduced radiation exposure, image manipulation, simple data storage, and rapid image display [61]. Endodontics places a premium on image quality because it permits precise interpretation of root and canal morphology [61]. Recent research has shown that ANNs can be used as a second opinion to improve the accuracy of WL determination by radiography and can function as a decision-making system in similar clinical scenarios [9]. The accuracy of ANNs was 93% [62], which is exceptional. In determining the apical foramen and working length [9], the AI-based model also demonstrated a high degree of accuracy of 96%. These models can be of great assistance to less experienced dentists and non-specialists, particularly in clinical settings where specialists are unavailable [63].

AI for Periapical Pathology Prediction

Clinical and radiographic evaluations may not always yield conclusive results, highlighting the need for dependable instruments to aid dentists in making precise treatment decisions. The use of AI technology for the diagnosis of periapical pathologies is on the rise [64]. An AI-based CNN model has been made to find periapical lesions on X-rays. It has shown better sensitivity and area under the receiver operating characteristic curve than oral and maxillofacial radiologists' interpretations [65]. The CNN model has the potential to aid dentists in the detection and diagnosis of periapical lesions, with satisfactory results in terms of high sensitivity and moderate specificity [66]. A separate research study [67] looked at the use of CBCT imaging, which is a 3D imaging technology that can accurately find periapical lesions and their size and location [67]. The analysis found that this model's reliability was 92% [67]. However, the results indicated that the model did not perform better than the reference standard, implying that future work could enhance the results through the application of optimization techniques [68].

AI for the Detection and Diagnosis of Vertical Root Fractures (VRFs)

VRFs are not common in endodontically treated teeth, but when they occur, they are considered severe complications that may lead to tooth extraction or root resection. VRF incidence ranges from 3.7% to 30.8% [69]. Detecting VRFs can be difficult, necessitating the development of alternative diagnostic techniques. Studies have demonstrated that CBCT imaging is superior to radiographs for detecting VRFs in unfilled teeth. However, radiographs detect VRFs in root-filled teeth marginally better. The use of AI technology has improved the diagnosis of VRFs [70]. Comparing CBCT images to periapical radiographs [67], probabilistic neural network (PNN) architecture has been found to be significantly more effective in diagnosing VRFs. Similarly, CNNs have been used with promising results to detect VRFs on orthopantomograms (OPGs), indicating that these models' accuracy and performance in detecting VRFs are very promising and can be extensively employed in clinical practice [66].

AI for Root Morphology Assessment

A thorough understanding of root and root canal system variations is essential for the efficacy of nonsurgical root canal treatment [54]. To aid in the identification of root morphologies on CBCT images, a CNN model with superior-quality results was devised [71]. In addition, a second study discovered that AI could perform at the same level as a human operator, but at a much quicker rate [30]. These models can serve as a beneficial instrument for accurate clinical diagnosis and the prevention of unanticipated situations [63]. The widespread use of AI technology in endodontics indicates that neural networks achieve results comparable to those of experts with greater accuracy and precision [63]. This is particularly true when diagnosing periapical pathosis, root fractures, determining working length, and predicting disease [54].

AI in Orthodontics

Utilizing facial photographs and orthodontic impressions, Thanthornwong [72] conducted a study to determine whether or not a patient needed orthodontic treatment. To develop a prediction model, they identified variables such as missing teeth, overjet, overbite, open bite, crossbite, and displacement. There were 1,000 participants in the research, and 80% of the data was used for training and 20% for assessment. Using a sample of 20 patients, the dataset was validated further. The model with the highest specificity [100%], sensitivity [95%], and accuracy [96%] was selected. Two orthodontists with more than five years of experience predicted the necessity of orthodontic treatment. The model was evaluated using data from 200 patients, with higher scores indicating the need for treatment and lower scores indicating no need for treatment.

The application of AI to orthodontic treatment planning

Over time, the use of AI in orthodontics for treatment planning and outcomes has garnered increasing attention [73,74]. Prior research involved the development of mathematical models to determine which patients required extractions. In a two-part investigation, researchers created a mathematical model to predict the necessity and desired pattern of extractions for a given case. The objective was to predict unanticipated treatment outcomes with extractions and correctly identify the characteristics that led to the selection of extractions. The model was loaded with patient data, including conventional photographs, radiographs, and orthodontic impressions. The model identified the characteristics of the presenting malocclusion and compared them to the system's closest prototype. Before the ultimate result was determined, multiple decisions were made based on the case's characteristics, and an aggregate calculation of the outcomes was performed. The accuracy of the model was evaluated against the decisions of clinicians, and a 90.4% accuracy rate was obtained. The crucial factor in obtaining a successful orthognathic surgery outcome is accurate diagnosis and treatment planning [75]. After identifying the patient's concerns and issues, the clinician must develop a comprehensive treatment plan to address the issues.

AI in Orthognathic Surgery

The process of preparing patients for orthognathic surgery can be protracted and complex, requiring both clinical and laboratory work. Acrylic splints are traditionally used as intraoperative guides, but these techniques are prone to error due to dimension changes and material fractures. To address these limitations [76], a set of surgical robotic limbs was created to transmit information from a virtual screen to the operating room and assist surgeons during the procedure. The mechanized arm has a 6-degree range of motion and can concentrate on specific tool center points. Condylar heads frequently shift during repositioning during mandibular surgery, resulting in post-surgical complications such as condylar sags. To prevent this, an electromagnetic sensor was developed to record the real-time movements of the condylar heads and provide 3D coronal and sagittal views to ascertain their position in the fossa [77]. On the other hand, AI has been studied for the creation of surgical prostheses [78]. Using scanning and CBCT images, researchers have created a 3D diagnostic model and a virtual orthodontic-orthognathic treatment plan. This resulted in the fabrication of a 3D prosthesis using 3D printing to serve as a surgical guide. AI can assist orthodontists in determining the optimal method to move teeth, but it does not currently account for oral diseases, facial analysis, or functional concerns when planning treatment. Nevertheless, AI has been incorporated into imaging diagnosis to enhance sensitivity and specificity for a wide spectrum of conditions, including the diagnosis of syndromes and the detection of caries [79].

AI for dental pathology

Maxillary Sinus Conditions

AI can be used to identify and diagnose pathological changes in the maxillary sinuses, which are typically seen on extraoral radiographs. For inexperienced dentists, this technology can help reduce the number of incorrect diagnoses and is a valuable tool. Kim et al. examined the diagnostic accuracy of an AI system by utilizing radiographs of the maxillary sinus in Water's view to examine the diagnostic accuracy of the system. The results demonstrated that the AI system had significantly greater sensitivity and specificity than radiologists. AI can aid in the identification of mucosal hypertrophy and retention cysts in the maxillary sinus, which are at times overlooked by radiologists. Kuwana et al. implemented a DL object detection technique. In another study, a CNN model was suggested to help radiologists find and divide mucosal hypertrophy and retention cysts from CBCT images [80,81].

Oral Cancer

Oral cancer is the sixth most prevalent form of cancer, and early diagnosis is essential for improved prognosis and survival rates. AI has the potential to enhance early diagnosis and reduce the morbidity and mortality associated with it. ANN was used by Nayak et al. to demonstrate the difference between normal, premalignant, and malignant tissues based on laser-induced autofluorescence spectra recordings. With an accuracy of 98.3%, a specificity of 100%, and a sensitivity of 96.5%, the method has the potential for real-time clinical applications [82]. Recent research on oral cancer has led to the successful development of AI models that can predict the occurrence and recurrence of this disease. The use of AI for the early detection of oral cancer is gaining popularity as a means of creating more precise and effective diagnostic instruments and enhancing patient care. AI-based applications that use clinical decision support systems for differential diagnosis of oral mucosal lesions can be helpful for screening, classifying suspicious changes in mucosal tissue, providing tissue diagnostics, predicting lymph node involvement, analyzing gene expression, and profiling the microbiota [83].

Salivary Gland Maladies

AI can help with the diagnosis of salivary gland diseases, which can be challenging for inexperienced dentists due to their similar morphologies. In some cases, DL models have demonstrated the ability to outpace radiologists. In a study conducted in Japan, DL was utilized to detect fatty degeneration of salivary gland parenchyma on CT scans, an indicator of Sjogren's syndrome. The investigation utilized 500 CT images, with 400 serving as training data and 100 serving as test data. The diagnostic performance of the DL system was comparable to that of experienced radiologists and substantially superior to that of less experienced radiologists [8]. Due to their uncommon occurrence and overlapping morphological characteristics, salivary gland tumors are difficult to diagnose. On the basis of their cytological appearance, ML has been used to identify malignant salivary gland tumors. 115 malignancy samples were classified into 12 morphological variables using a recursive partitioning algorithm, and the performance was compared to that of an experienced clinician. The decision tree system test can effectively limit the differential diagnosis and enhance pathological diagnosis accuracy. AI is also capable of predicting the recurrence of salivary gland cancers. Chiesa-Estomba et al. utilized clinical, radiological, histological, and cytological data to predict the occurrence of facial nerve dysfunction in patients undergoing surgical treatment of salivary gland tumors accompanied by posterior nerve injury. AI could be used as a diagnostic tool to predict facial nerve injury, allowing surgeons and patients to be aware of potential complications beforehand [84].

Dental diagnosis requires accurate data interpretation, and standardizing and comparing datasets may improve the accuracy of AI models in identifying dental caries, VRFs, and predicting dental restoration failures. Access to public data sets can also aid in the creation of AI models.

Implementing AI in healthcare does not come without obstacles. AI has tremendous potential in healthcare, but there are technical and ethical obstacles that must be overcome. The development of AI-based systems is led by computer scientists without medical training, resulting in a problem-centric approach to healthcare delivery [84]. AI cannot replace current healthcare models that rely on clinician expertise and patient communication, and the use of robotic assistants has raised questions [85]. Additionally, dental professionals are reluctant to implement AI technologies.

AI has several advantages in dentistry, including the ability to perform tasks quickly and make accurate diagnoses based on logical and feasible decisions. AI can also optimize procedure standardization. Nonetheless, there are also some drawbacks to consider. The mechanism or system's complexity necessitates a learning curve, and implementation can be costly. In addition, the systems require adequate instruction and require data for both training and testing. The results of an AI procedure may not be immediately applicable. Through CT medical images [86], AI models have been beneficial in detecting and diagnosing COVID-19. The face recognition system is another technological advancement in the field of AI. AI-based technologies are now transforming from being considered a scourge to a benefit, and they are impacting every aspect of society, healthcare, and politics, including dentistry [87].

Although AI is becoming more prevalent in dentistry as a result of technological advancements and digitization, dental professionals should be wary of relying solely on AI for diagnosis and treatment decisions. Notwithstanding, NNS can improve diagnostic precision, speed, and efficacy [87].

Dentistry has recently been utilizing ChatGPT, a chat-generative pre-trained transformer. This computer program uses AI and has been trained on enormous quantities of data to generate responses to user inputs that imitate human-like language. This technology seeks to improve the communication skills, language processing, and responsiveness of computers through text-based interfaces using ML and DL techniques [88-92].

ChatGPT has the potential to provide a variety of services to a variety of groups, including educators, healthcare professionals, and patients. Students can profit from this technology by obtaining assistance with schoolwork and tutoring due to its capacity to respond to questions and clarify complex concepts. In addition, it has the potential to revolutionize the way biomedical science is taught as a teaching instrument [90,91].

ChatGPT is a useful resource for patients, especially those undergoing surgery, as it can provide information and education prior to and following the procedure. This technology can address medical queries and give patients realistic surgical outcome expectations [87,89]. The implementation of ChatGPT in healthcare and dentistry can have a considerable positive impact on patient empowerment, independence, service efficacy, and safety. In addition, it can contribute to the expansion of access to and the improvement of the quality of care, as well as the empowerment and facilitation of patients.

Despite its potential advantages, ChatGPT has limitations that are characteristic of any new technological innovation. Involving a large-scale neural network that necessitates a substantial amount of computational power and memory contributes significantly to the high price. This may present difficulties for minor medical applications with limited resources. ChatGPT cannot incorporate external information sources, which can reduce the system's precision in the medical industry. It is important to remember that it does not always provide accurate references or any at all; answers may vary depending on the question and preceding conversation or context; and it may provide incorrect answers that sound convincing, which is a problem that must be addressed so that ChatGPT can appropriately acknowledge uncertainty [89].

The introduction of advanced dental technologies and specialized AI in dentistry has ushered in a new era known as Dentronics. This new era may offer several advantages, including enhanced dependability, reproducibility, precision, and efficacy in dental practices. In addition, dentronics has the potential to improve our understanding of disease pathogenesis, risk assessment strategies, diagnoses, and disease prognosis, resulting in improved treatment outcomes. It is not fiction that AI is becoming increasingly prevalent in the field of dentistry. Despite the fact that AI cannot replace dentists because dental practice involves more than just disease diagnosis, including the correlation of clinical findings and the administration of treatment to patients, a thorough understanding of AI concepts and techniques will be advantageous in the future [90].

## Conclusions

Limitations and challenges of AI in dentistry

Various sources discuss the limitations and challenges of AI in dentistry. The need for large datasets to train AI models, the possibility of bias in the data used for training, and the inability of AI systems to fully replace human judgment and expertise are some of the limitations. In addition, the article discusses obstacles including the ethical implications of AI decision-making, the need for regulatory frameworks, and the potential impact on the dentist-patient relationship. Integrating AI systems into dental practice can be challenging due to the initial investment costs and the need for dentists to obtain new skills for working with AI-based technologies.

Conclusion

AI models have the potential to aid in the diagnosis of dental lesions and vertical tooth fractures, as well as detect the tooth's finishing line and predict the likelihood of restoration failure. AI is anticipated to revolutionize dentistry and make dental professionals' tasks simpler and more accurate. It is anticipated that as technology advances, AI will become the new standard in dentistry and help professionals perform their duties more efficiently while reducing human error. Currently, the most significant barrier to implementing AI in dentistry is the lack of adequate and accurate data. As a result, dentists and clinicians must concentrate on gathering and entering accurate data into their databases, which AI will use in the field of dentistry in the near future. AI models for dental applications are still in development. Further research is required to investigate the potential of neural networks in dentistry and to incorporate them into routine dental practice.
